# Polymeric Nano-Micelles as Novel Cargo-Carriers for LY2157299 Liver Cancer Cells Delivery

**DOI:** 10.3390/ijms19030748

**Published:** 2018-03-06

**Authors:** Nemany Abdelhamid Nemany Hanafy, Alessandra Quarta, Marzia Maria Ferraro, Luciana Dini, Concetta Nobile, Maria Luisa De Giorgi, Sonia Carallo, Cinzia Citti, Antonio Gaballo, Giuseppe Cannazza, Rosaria Rinaldi, Gianluigi Giannelli, Stefano Leporatti

**Affiliations:** 1 CNR NANOTEC-Istituto di Nanotecnologia, Via Monteroni, 73100 Lecce, Italy; 2 Department of Mathematics and Physics “E. De Giorgi”, University of Salento, Via Monteroni, 73100 Lecce, Italy; 3 Department of Biological and Environmental Sciences and Technologies (DiSTeBA), University of Salento, Via Monteroni, 73100 Lecce, Italy; 4 Life Science Department, University of Modena e Reggio Emilia, Via G. Campi, 41125 Modena, Italy; 5 National Institute of Gastroenterology “S. De Bellis”, IRCCS Research Hospital, Via Turi, 70013 Castellana Grotte (BA), Italy

**Keywords:** nano-micelles, Cargo-carriers, LY2157299, Liver Cancer Cells, drug delivery

## Abstract

LY2157299 (LY), which is very small molecule bringing high cancer diffusion, is a pathway antagonist against TGFβ. LY dosage can be diluted by blood plasma, can be captured by immune system or it might be dissolved during digestion in gastrointestinal tract. The aim of our study is to optimize a “nano-elastic” carrier to avoid acidic pH of gastrointestinal tract, colon alkaline pH, and anti-immune recognition. Polygalacturonic acid (PgA) is not degradable in the gastrointestinal tract due to its insolubility at acidic pH. To avoid PgA solubility in the colon, we have designed its conjugation with Polyacrylic acid (PAA). PgA-PAA conjugation has enhanced their potential use for oral and injected dosage. Following these pre-requisites, novel polymeric nano-micelles derived from PgA-PAA conjugation and loading LY2157299 are developed and characterized. Efficacy, uptake and targeting against a hepatocellular carcinoma cell line (HLF) have also been demonstrated.

## 1. Introduction

Encapsulation of cancer drugs in a single carrier has become one of interesting topic, especially for those drugs that have an inhibitor effect on signaling pathways. The toxicity of cancer therapy can cause major complications on normal cells, such as low white-blood-cell counts or heart failure that necessitate cessation of treatment [1] and might impair the physiological functions of other organ. The main challenges that are associated with existing cancer treatments are principally related to how therapy can be localized into tumor sites.

Polymeric micelles assembled by hydrophobic–hydrophilic polymers have proved to be highly effective drug delivery vehicles [2,3,4,5,6], because they increase drug solubility, reduce toxicity, increase circulation time, enhance tissue penetration, and have targeting ability [7,8,9,10]. Fabrication of nano-micelles was assessed on fact that the mechanical properties of poly galacturonic acid (PgA) can be modified by combination with a second polymer [11]. Besides, PgA is not able to be degraded in the upper gastrointestinal tract due to its insolubility at acidic pH and is high soluble in colon. Hence, PgA-PAA micelles might cross GI with no destruction. After that, PgA-PAA micelles will be absorbed into the blood stream from through small intestine. Nano micelles will be driven into liver cells through blood stream. The strategy of this study is to avoid PgA solubility in the colon by its combination with polyacrylic acid (PAA). It is thought that inhibition of the signaling pathway of TGFβ might create potential therapeutic agent in modern cancer treatment [12]. Among many TGFβ inhibitors that have used previously [13] against hepatocellular carcinoma cell line (specifically HLF) there is LY2157299 (LY), a very small molecule having high tumor diffusion. It can block signalling through the heteromeric TGFβ receptor complex to reduce the levels of active phosphorylated small mother against decapentaplegic (SMAD) [14]. The issue of its dosage is that it can be diluted by blood plasma, engulfed by immune system or it might be lost during digestion in gastrointestinal tract. Additionally, it can induce resistance by cancer cells. The oral routine is the most accepted methods among many dosage administrations because of its simplicity, convenience, and patient acceptance, especially in the case of repeated dosing for chronic therapy. For this reason our aim was to optimize “nano-elastic” carriers having ability to avoid acidic pH of upper gastrointestinal tract, alkaline pH of colon, anti-immune recognition, targeted for cancer cells, having high diffusion, and high drug capacity. The second goal was to encapsulate LY and to investigate changes of Golgi apparatus morphology after inhibition of TGFβ1, since it is known that Golgi apparatus is the main TGFβ1 pro-peptide [15]. This second part will be the subject of a future follow-up article.

## 2. Results

### 2.1. Physical Properties of PgA and PAA

**Figure 1:**
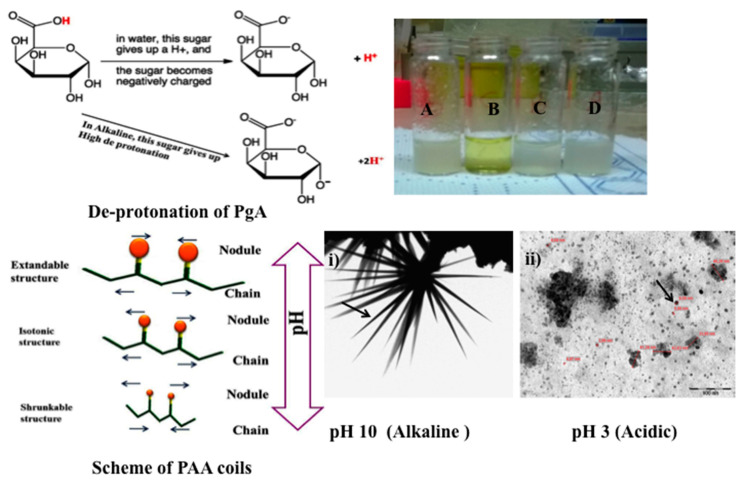
(**Left**) scheme of polygalacturonic acid (PgA) de-protonation and scheme of poly (acrylic) acid (PAA) coils; (**Right**): PgA at different pH; (A) Distilled water; (B) pH 10; (C) pH 7.2; (D) pH 3. TEM images of (i) PAA at alkaline pH and (ii) PAA at acidic pH. (Arrows indicate different nanoparticles shape).

Polygalacturonic acid (PgA) is a natural polymer having a heterogeneous structure that is bonded via α (1 → 4) glycosidic linkage. PgA has limited dissolution at both distilled water, PBS (pH: 7.3), and acidic distilled water (pH: 3), resulting in white turbidity (e.g., see Figure 1A,C,D). This result is compatible with its hydrophobic properties [16,17]. In this sense, PgA is not degraded in upper gastrointestinal tract due to its insolubility in acidic condition [18]. Contrarily, PgA can be dissolved in alkaline distilled water (pH 10), forming clear yellow greenish color (see Figure 1B). In this case, polyacrylic acid (PAA) could be an interesting polymer to be combined with PgA.

Poly (acrylic) acid (PAA) is a hydrophilic polymer [19], with random coils on its chains consisting of only thermal blobs. These coils have swelling properties under ionic and salt strength that lead to extend its chain in alkaline solution (e.g., see scheme of PAA coils) [20]. The extendable and shrinkable properties at alkaline and acidic pH (for example see Figure 1i,ii) are the main physical properties of PAA (e.g., see Figure 1i,ii) [20,21,22,23]. The reason of these properties is due to its hydrogel PAA compounds that can answer to pH changes [24,25,26]. In hydrated media of appropriate pH and ionic strength, the carboxylic groups develop and ionize fixed charges on the polymeric network, thus producing electrostatic repulsive forces that are responsible for pH-dependent swelling or de-swelling of the hydrogel structure [27,28].

### 2.2. Synthesis of Nano-Micelles

The presence of free hydroxyl and carboxyl groups that are inserted in both PgA and PAA chains can increase the accessibility for ionization at alkaline pH. This is due to deprotonation of their hydrogen atoms under ionic strength (e.g., see in Figure 1 scheme of de-protonation of PgA). Hence, PgA and PAA are expected to have crosslinking structure due to the esterification of PgA. The strategy of this study focused on avoiding the solubility of the polygalacturonic acid in the colon, which has an alkaline pH. This strategy can occur by complexing PgA with other biopolymers [11,29], like PAA. Thus hydrogel PAA nodules have swelling properties in aqueous media due to presence of carboxyl groups on their chain, which are strongly associated with water molecules. These groups are readily ionizable and are sensitive to the effects of pH and ionic strength [30], resulting in a large osmotic swelling force. These properties have raised value of PgA functionalized by PAA. In this sense, PgA-PAA complex can swell at alkaline pH covering PgA inside, causing a good stabilizer for PgA against alkaline condition of colon. Perhaps their cross-linked nature may cause modification of PgA chain and it can prevent hydrogen protonation. Additionally, PAA is poorly soluble in water at low pH, causing a shrinkable structure (e.g., see Figure 1ii). This mechanical elasticity perhaps causes supporting for micelles against the oral and gastro intestinal tract [31].

### 2.3. LY2157299 Loaded Nano-Micelles

**Figure 2:**
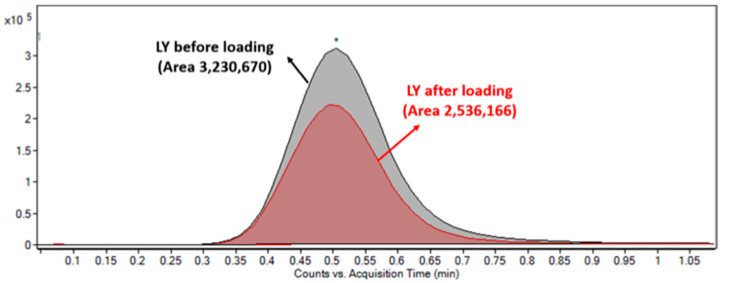
Measurement of LY2157299 (LY) encapsulated by mass spectrometry: overlapping chromatograms of EIC m/z 370.1664 before (black peak) and after (red peak) loading. The peak obtained after loading refers to LY concentration in the supernatant after centrifugation. *: LY concentration Peak in the supernatant; Y-axis: number of Counts.

The main reaction occurring between LY2157299 and moieties of micelles is amino-carboxyl electrostatic interaction. Hence, LY2157299 molecules can pass into the core of micelles that is driven by osmotic pressure and thanks to the permeability of pores that are produced by 40 °C temperature. Loaded LY2157299 might react with negative charge of micelles moieties, keeping LY2157299 inside. After centrifugation, the unloaded LY2157299 was quantified in the supernatant by using mass spectrometry (see Figure 2). The loading efficiency was 23%.

### 2.4. Characterization

**Figure 3:**
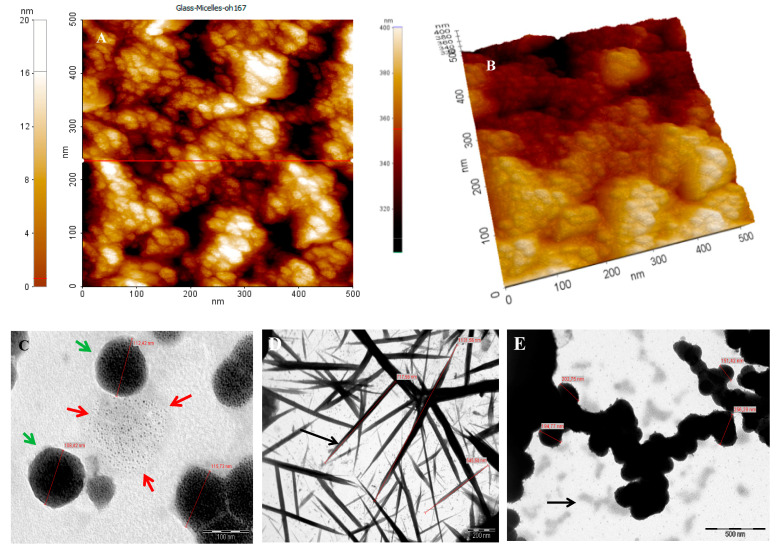
Characterization of nano-micelles. (**A**) Topographical Atomic Force Microscopy (AFM) image of nano-micelles; (**B**) AFM 3D visualization of nano-micelles; (**C**) High magnification Transmission Electron Microscopy (TEM) image of details of nano-micelles; (**D**) TEM image of extended nano-micelles; (**E**) TEM image of shrunk nano-micelles.

**Figure 4:**
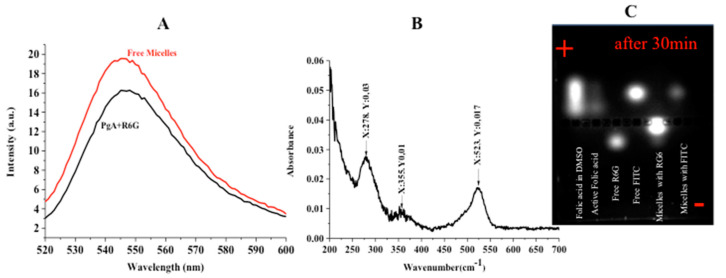
Measurement of nano-micelles markers. (**A**) Fluorescence spectrophotometry; (**B**) Absorbance spectrophotometry; (**C**) Agarose Gel separation (after 30 min).

The so fabricated micelles appeared to have spherical shapes, with diameters ranging from 100–200 nm. Their surfaces consist of many dark structures like spots (See Figure 3C (green arrows)). Also, micelles that were under formation have many dark spots collected together (see Figure 3C (red arrows)). This reveals that the internal and external structure of micelles consist of several collected spots having internal cross-linked structure. Topography of micelles refers to many small aggregated spots on surface (e.g., see Figure 3C,D). In agreement to previous reports [32,33], PAA is expected to form hydrophilic corona of PgA-PAA complex. In this case, the swelling of PAA polymer on water makes it favourable for protection because PAA adsorbs water many more times than its weight in alkaline pH [34]. The UV-Visible absorption spectrum indicated that the absorption peaks of folate-micelles at 278 and 355 nm are assigned to folate peaks (e.g., see Figure 4B). This confirms the covalent attachment of the folic acid according to previous reports [35]. However absorption peak at 523 nm reveals R6G conjugation. The successful attachment of PgA-R6G was investigated by using fluorescence spectrophotometry (see Figure 4A). Hence, the amino group of R6G is attached electrostatically by carboxylic group of PgA. This property was used for assembled micelles structure having fluorescence intensity at the same wavelength of PgA-R6G. Micelles moieties that are run on agarose gel showed strong attachment, even to 30 min., running under 60 V (e.g., Figure 4C). In this sense, R6G labeled micelles were separated more difficulty when compared to micelles that are labeled by Fluorescein isothiocyanate (FITC) (see Figure 4C).

### 2.5. FTIR Characterization, Folic Acid Conjugation and Structure Assembly

**Figure 5:**
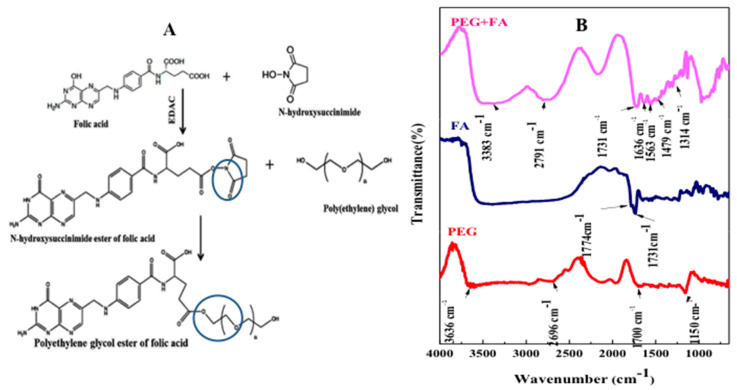
Folic acid conjugated with Poly (ethylene) glycol. (**A**) Scheme of FA-PEG conjugation; (**B**) FTIR spectra of PEG, PEG+FA, FA (arrows indicate selected bands).

Folic acid (FA) is a well known ligand used widely for tumor targeting due to its high binding affinity for foliate receptor on cancer cell membrane [36]. In this study FA was dissolved by using dimethyl sulfoxide (DMSO) [37], then its carboxyl group was activated by *N*-hydroxy succinimide (NHS) forming esters bond [38]. In the presence of *N*-Ethyl-*N*′-(3-dimethylaminopropyl) carbodiimide hydrochloride (EDAC), FA–COOH was cleaved and its carboxyl group is joined to oxygen of NHS by single bond forming N-hydroxy succinimide ester of folic acid (see Scheme S1). This structure was modified by PEG-hydroxyl group forming folic acid ester of PEG and dicyclohexyl urea that was removed by dialysis bag (See Figure 5, and Scheme S1). In agreement to these results, hydroxyl group joining carboxyl group was also performed successfully by Nair et al. [16]. However in the presence of EDAC, hydroxyl group of poly (d-valerolactone)/poly (ethylene glycol)/poly (dvalerolactone) (VEV) copolymer was grafted with carboxylic group of folic acid.Furthermore hydroxyl group of PEG can be easily modified by aliphatic chains molecules or small amino-acids [38].

In Figure 5B the location of 1774 cm^−1^ band at FTIR spectrum of activated FA corresponds to the stretching bond of C=O and 1731 cm^−1^ band is related to the acrylate group of C=C indicating formation of vinylic linkage [39]. This result confirms that carboxyl group of folic acid was esterified by hydroxyl group of N-hydroxy succinimide. In the FA-PEG spectrum, carboxylic FA group is accompanied by the appearance of two bands in the 1563–1636 cm^−1^ due to νs(COO^−^) and bands at range 1479–1314  cm^−1^ assigned to νs(COO^−^). Frequencies of the bond orders of both C=O bonds can be changed according to type of reaction. The broad stretching vibration of OH− group ν(O–H) occurred at ∼3383 cm^−1^.

**Figure 6:**
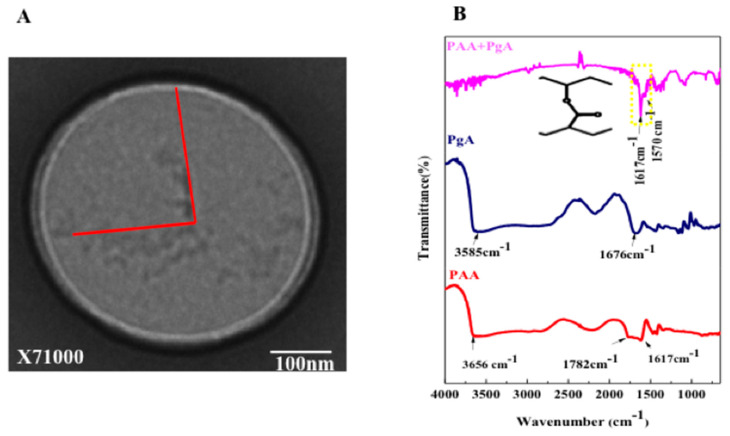
Characterization of Polygalacturonic acid (PgA) esterified Polyacrylic acid (PAA) nano-micelles. (**A**, radial arrows indicate round-shaped micelle) TEM image of a nano-micelle and (**B**) FTIR spectra of PGA/PAA nano-micelles conjugation.

In Figure 6B FTIR spectrum of PAA shows the typical bands for carboxylic acids, with the stretching absorption associated with the hydroxyl groups (O–H) in 3656 cm^−1^, the two bands at 1782 and 1617 cm^−1^ are assigned to vibrations of the O=C and C=C structure [40]. Free PgA spectrum shows that 3585 cm^−1^ band is due to O–H stretching vibration, while the carbon-oxygen (C=O) absorption peak was observed at 1676 cm^−1^ [41]. Spectrum of PgA-PAA showed strong absorption assigned to carbonyl group (C=O) at 1617 cm^−1^ and C=C at 1570 cm^−1^. In Figure 6A TEM images confirmed that PAA and PgA were assembled as spherical layers composited in one single system.

**Figure 7:**
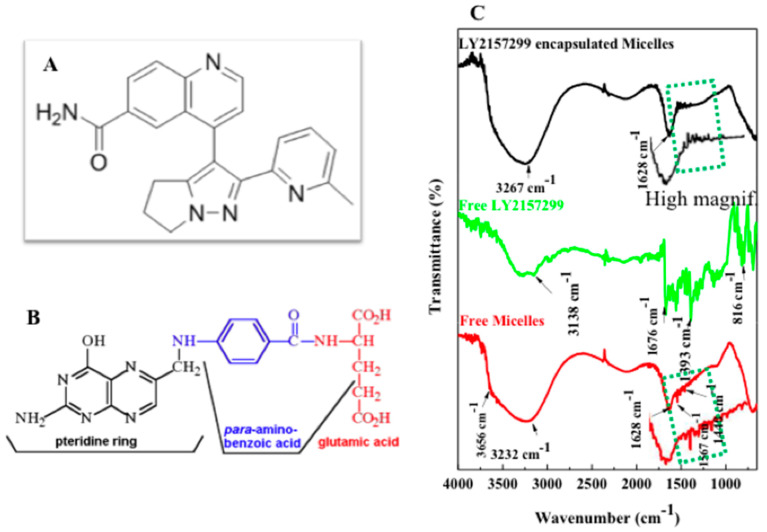
Bands Modification of Encapsulated LY2157299. (**A**) LY215299 structure; (**B**) Folic acid structure; (**C**) FTIR spectra of LY/LYfree micelles and LY-loaded micelles (squared dotted lines refer to selected bands).

In Figure 7C FTIR spectrum of free micelles-FA shows a broad absorption between at 3656 cm^−1^ and 3232 cm^−1^ that corresponds to O–H and N–H stretching vibrations. This indicates that strong inter-molecular and intra-molecular hydrigen bonding is obtained [42] between PgA-PAA and PEG-FA. Aromatic ring stretch of the pyridine and p-amino benzoic acid moieties can be easily referred to different small peaks (down direction) [43] that they are measured within the range of 1440–1628 cm^−1^. FTIR of LY2157299 spectrum shows that 3138 cm^−1^ band is related to stretching vibration of amide I (NH). Bands between 1676 cm^−1^ and 1393 cm^−1^ refer to pteridine ring vibration. 816 cm^−1^ band is related to aromatic ring structure. FTIR spectrum of encapsulated LY2157299 shows wide adsorption at 3267 cm assigned to stretching vibration of overlapping O–H, N–H and H-aromatic structure. This indicates that there is strong connection formed also after LY2157299 encapsulation. The characteristic bands of the pyridine and p-amino benzoic acids of folic acid were clearly showed as small peaks (up direction) viceversas to those that are located at free micelles.

### 2.6. Cell Cultures and Micelles Uptake

**Figure 8:**
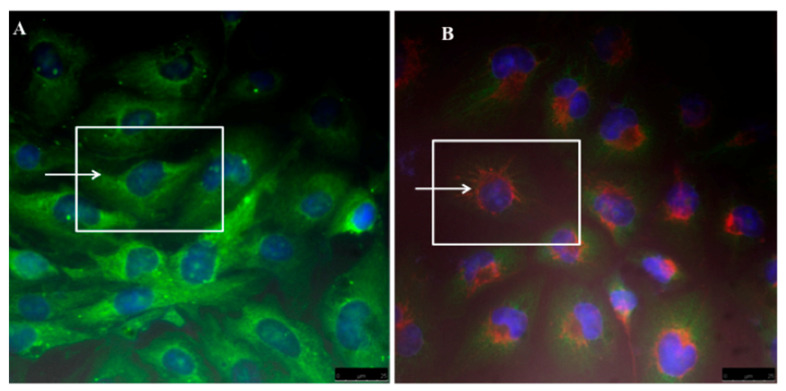
Confocal laser scanning fluorescence microscopy images of SMA (green) and Micelles (Red). (**A**) Control HLF (without LY-loaded micelles); (**B**) HLF incubated with encapsulated LY. (Scale bar: 25 µm) (Arrows and square indicate nanomicelles uptake).

The cellular internalization of micelles was measured by confocal laser scanning fluorescence microscopy (for details see Supplementary Materials). Hence R6G labelled micelles were successfully localized inside the cytoplasm as demonstrated by fluorescence emission of red colour located in the perinuclear region compared to control hepatocellular carcinoma cell line (specifically HLF) whilst green colour is assigned to Alexa-Flour -anti-mouse fragmentation used to detect αSMA (e.g., see Figure 8). Fluorescence images clearly demonstrate that nano-micelles were readily internalised in cellular compartments and this allows successful drug release against liver cancer cells.

## 3. Discussion

Polygalacturonic acid (PgA) is a natural polymer with limited dissolution in water. In fact, PgA is not degradable in upper gastrointestinal tract due to its insolubility in acidic condition [18] but can be dissolved in alkaline distilled water (pH 10). The reason of this dissolution is related to its carboxyl groups that are partially in the methyl ester structure with different degree of esterification (DE) and amidation (DA). For instance, acidity condition can raise DE of pectin causing hydrophobicity. When DE reached to less than 50%, pectin is highly water-soluble [11]. It is reported that combination of PgA with a second polymer into a composite may alter the degree of swelling and can change its mechanical properties [11], improving in the most cases the stability of drugs and controlling the drug release. The strategy used in this study is to combine PgA with polyacrylic acid (PAA), a hydrophilic polymer [19] with random coils having swelling properties under ionic and salt strengths leading to extend its chain in alkaline or to shrink in acidic solution. These properties are related to the hydrogel nature of PAA able to react upon pH changes [24,25,26]. Furthermore in aqueous solution electrostatic forces are generated, causing swelling or de-swelling of hydrogel [27,28]. Thus hydrogel PAA swelling properties are due to presence of carboxyl groups on their chains. These groups are readily ionizable and sensitive to the effects of pH and ionic strength [30], causing a large osmotic swelling force. Our strategy is therefore to conjugate PgA with PAA forming a complex PgA-PAA that can swell at alkaline pH being a good stabilizer for PgA against alkaline condition of colon. On the other side PAA is poorly soluble in water at low pH and its mechanical elasticity could be a support to produce nano-micelles against oral and gastro-intestinal tract [31]. Upon increasing temperature up to 40 °C an inihbitor of TGFβ (LY2157299) was successfully -and with relatively high efficiency (23%)-loaded inside hybrid PgA-PAA nano-micelles by passing into their core driven by osmotic pressure and permeability of their pores. Spherical shaped nano-micelles with nano-sized aggregated spots on surface were produced. As reported earlier [32,33], PAA formed hydrophilic corona of PgA-PAA complex. Hence, swelling of PAA polymer on water can result in protection since PAA adsorbs more water than its weight in alkaline pH [34]. UV-Visible absorption spectrum confirms the covalent attachment of the Folic Acid (FA) according to previous reports [35] and reveals also Rhodamine conjugation. Folic acid (FA) is a well-known ligand used broadly for tumor targeting due to its high binding affinity for folate receptors on cancer cell membrane [36]. FA was dissolved by using dimethyl sulfoxide (DMSO) [37], then its carboxyl group was activated by N-hydroxy succinimide (NHS) causing esters bond [38]. FTIR spectroscopy has been used to investigate appearance of bonds during\after FA functionalization of PgA-PAA nano-micelles. Results confirm that carboxyl group of folic acid was esterified by hydroxyl group of N-hydroxy succinimide. In fact 1774 cm^−1^ band at FTIR spectrum of activated FA corresponds to the stretching bond of C=O and 1731 cm^−1^ band relates to the acrylate group of C=C indicating a vinylic linkage [39]. The two bands at 1782 and 1617 cm^−1^ are assigned to vibrations of the O=C and C=C structure [40]. PgA spectrum 3585 cm^−1^ band is due to O–H stretching vibration, whereas C=O absorption peak was observed at 1676 cm^−1^ [41]. Furthermore TEM images showed that PAA and PgA were assembled as spherical layers in one single system. Moreover FTIR spectrum of free micelles-FA demonstrates production of a strong inter-molecular and intra-molecular H-bonding [42] between PgA-PAA and PEG-FA.

However FTIR spectrum of loaded LY2157299 evidences overlapping O–H, N–H and H-aromatic structure resulting in a strong connection formed. Finally fluorescence microscopy investigation clearly demonstrates that labelled nano-micelles were successfully localized and internalised inside the cytoplasm envisaging a successful drug release against liver cancer cells. According to previous reports and these results, it is believed that PAA cross-linked PgA may offer good stabilizer against pectin degradation at alkaline pH of colon. Furthermore, physical properties of PgA could be modified by conjugation with PAA.TEM confirms PAA and PgA were assembled as spherical layers composited in one single nano-system. FTIR of PgA-PAA spectrum shows strong absorption assigned to carbonyl attachment of PgA-PAA. Their separation by agarose gel confirms that they have strong attachment and good stability. Moreover, PgA-PAA micelles have the same properties of PAA alone in alkaline and acidic condition. Hence, they are shrunk and extended upon change of pH. The “nano-elastic” properties of PgA-PAA micelles have strengthened their potential use for oral and injected dosage.

## 4. Materials and Methods

### 4.1. Chemicals

The suppliers of the chemicals were as follows: poly (galacturonic acid) (PGA) was purchased from Fluka-Sigma Aldrich, St. Louis, MO, USA; LY2157299 from Eli Lilly company, Indianapolis, IN, USA; PBS pH 7.3 was purchased from Oxoid Limited Basingstoke, Hampshire, England; Ethanol from Baker Analyzed, Fisher Scientific, Landsmeer The Netherlands; Poly (acrylic acid) sodium salt (PAA); Poly(ethylene glycol) (PEG); Folic acid (FA); 2-(4-Amidinophenyl)-6-indolecarbamidine dihydrochloride, 4′,6-Diamidino-2-phenylindole dihydrochloride (DAPI); Paraformaldehyde, *N*-Ethyl-*N*′-(3-dimethylaminopropyl)carbodiimide hydrochloride (EDAC), *N*-hydroxy succinimide (NHS), Dimethyl sulfoxide (DMSO) from Sigma-aldrich, St. Louis, MO, USA and Anti-mouse IgG (H + L), F(ab’)2 Fragment (Alexa Fluor^®^ 488 Conjugate) #4408 were purchased from Cell Signalling Technology, Danvers, MA, USA.

### 4.2. Fabrication of Nano-Micelles

Polymers preparation, labeling, conjugation of folic acid with polyethylene glycol, Folic acid activation, esterification, quantification of LY2157299 loaded nano-micelles by using Liquid chromatography coupled to mass Spectrometry (LC-MS) and characterisation are described in details in Supplementary Material. 5 mL of PgA-R6G and 4 mL of PAA were mixed inside glass vial for 15 min. rotation by using magnetic starrier (1400 power) then 5 mL of PEG-FA was added. After that magnetic starrier rotation was completed in 30 min. The final product was centrifuged at 13,000 rpm for 1 h. The supernatant was discarded and PBS at pH 7.3 was added. Micelles were incubated in cold room until used. Further details are available in Supplementary Material.

### 4.3. Transmission Electron Microscopy (TEM)

10 µL of micelles suspension was deposited on the copper grid and air- dried before measurement. Copper grids sputtered with carbon films were used to support the sample. High-resolution TEM images of nanomicelles (Figure 6 and Scheme S1B) were analyzed by a Hitachi HT 7700 operating at 100 kV, coupled with a GATAN camera ORIUS SC600 with a resolution of 7 Megapixel. The GATAN camera is controlled by Digital Micrograph.

### 4.4. Atomic Force Microscopy (AFM)

10 µL of micelles suspension was dropped onto surface of clean glass. They were dried overnight and their morphological structure and diameter (e.g., about 500 nm) was measured by using AFM. Non-contact images were acquired by XE100 (AFM Park-Gambetti). The surface of a clean glass was used as background reference.

### 4.5. Absorbance and Fluorescence Spectrophotometry

The absorbance of folic acid was measured by using 300 Cary Eclipse UV Absorbance Spectrophotometer in special 1 mL Agilent Cary Eclipse cuvette. 1 mL of fabricated micelles was scanned at range 200–600 nm. Also, R6G conjugated micelles was measured by fluorescence spectrophotometer by the same procedure. The results were analysed by Origin 8.

### 4.6. Fourier Transform Infrared Spectroscopy (FTIR)

FTIR experiments were carried out by using JASCO Fourier Transform Infrared Spectrometer (FT/IR-6300) to detect the surface molecular structures in the range of 500–4000 cm^−1^. 10 µL of each sample (activated FA, PEG, and PEG-FA) and (PgA, PAA, PEG-FA and free micelles) was dropped onto clean silicon wafer substrates separately and was allowed to dry overnight. For all of the tests, at least three scans were recorded on different regions on the samples and representative spectra were analysed. Silicon wafer plate was used to collect the background spectrum. The comparison of interest area was detected in the range of 650–4000 cm^−1^. Further details about Confocal Microscopy and Scanning Electron Microscopy are available in the Supplementary Material.

## 5. Conclusions

In this paper, novel biodegradable polyacrylic\polygalacturonic acid hybrid nano-micelles have been synthetized and characterized for the loading of LY2157299, which is an inhibitor of TGFβ to treat hepatocellular carcinoma cancer cells. A new “nano-elastic” carrier that is able to avoid acidic PH of gastrointestinal tract, colon alkaline pH, and anti-immune recognition has been developed. Efficacy, uptake, and targeting against a hepatocellular carcinoma cell line (specifically HLF) have been demonstrated, paving the way for orally and injecting dose in clinical and preclinical (potential) use.

## Supplementary Materials

Supplementary materials can be found at https://www.mdpi.com/1422-0067/19/3/748/s1.

## References

[1] Chow E.K., Ho D. (2013). Cancer Nanomedicine: From Drug Delivery to Imaging. Sci. Transl. Med..

[2] Zhang X., Burt H.M., Mangold G., Dexter D., Von Hoff D., Mayer L., Hunter W.L. (1997). Anti-tumour efficacy and bio-distribution of intravenous polymeric micellar paclitaxel. Anticancer Drugs.

[3] Shin I.G., Kim S.Y., Lee Y.M., Cho C.S., Sung Y.K. (1998). Methoxy Poly(ehylene glycol)/ε-Caprolactone Amphiphilic Block Copolymeric Micelle containing Indomethacin. I. Preparation and characterization. J. Control Release.

[4] Yu B.G., Okano T., Kataoka K., Sardari S., Kwon G.S. (1998). In vitro dissociation of antifungal efficacy and toxicity for amphotericin B-loaded poly(ethylene oxide)-block-poly(beta-benzyl-l-aspartate) micelles. J. Control Release.

[5] Jeong Y.L., Nah J.W., Lee H.C., Kim S.H., Cho C.S. (1999). Adriamycin release from flower-type polymeric micelle based on star-block copolymer composed of poly(gamma-benzyl-l-glutamate) as the hydrophobic part and poly(ethylene oxide) as the hydrophilic part. Int. J. Pharm..

[6] Hanafy N.A., Dini L., Citti C., Cannazza G., Leporatti S. (2018). Inhibition of Glycolysis by Using a Micro/Nano-Lipid Bromopyruvic Chitosan Carrier as a Promising Tool to Improve Treatment of Hepatocellular Carcinoma. Nanomaterials.

[7] Nishiyama N., Kataoka K. (2006). Current state, achievements, and future prospects of polymeric micelles as nanocarriers for drug and gene delivery. Pharmacol. Ther..

[8] Hanafy N.A.N., Quarta A., Di Corato R., Dini L., Nobile C., Tasco V., Carallo S., Cascione M., Malfettone A., Soukupova J. (2017). Hybrid polymeric-protein nano-carriers (HPPNC) for targeted delivery of TGFβ inhibitors to hepatocellular carcinoma cells. J. Mater. Sci. Mater. Med..

[9] Torchilin V. (2009). Multifunctional and stimuli-sensitive pharmaceutical nanocarriers. Eur. J. Pharm. Biopharm..

[10] Zhang Y., Li Q., Welsh W.J., Moghe P.V., Uhrich K.E. (2016). Micellar and structural stability of nanoscale amphiphilic polymers: Implications for anti-atherosclerotic bioactivity. Biomaterials.

[11] Liu L., Fishman M.L., Kost J., Hicks K.B. (2003). Pectin-based systems for colon-specific drug delivery via oral route. Biomaterials.

[12] Gueorguieva I., Cleverly A.L., Stauber A., Sada Pillay N., Rodon J.A., Miles C.P., Yingling J.M., Lahn M.M. (2014). Defining a therapeutic window for the novel TGF-β inhibitor LY2157299 monohydrate based on a pharmacokinetic/pharmacodynamic model. Br. J. Clin. Pharmacol..

[13] Hanafy N.A., Ferraro M.M., Gaballo A., Dini L., Tasco V., Nobile C., De Giorgi M.L., Carallo S., Rinaldi R., Leporatti S. (2016). Fabrication and Characterization of ALK1fc-Loaded Fluoro-Magnetic Nanoparticles Rods for Inhibiting TGF β1 in HCC. RSC Adv..

[14] Bueno L., de Alwis D.P., Pitou C., Yingling J., Lahn M., Glatt S., Trocóniz I.F. (2008). Semi-mechanistic modelling of the tumour growth inhibitory effects of LY2157299, a new type I receptor TGF-beta kinase antagonist, in mice. Eur. J. Cancer.

[15] Taipale J., Miyazono K., Heldin C.H., Keski-Oja J. (1994). Latent transforming growth factor beta 1 associates to fibroblast extracellular matrix via latent TGF-beta binding protein. J. Cell Biol..

[16] Nair K.L., Jagadeeshan S., Nair S.A., Kumar G.S.V. (2013). Folic Acid Conjugated δ-Valerolactone-Poly(ethylene glycol) Based Triblock Copolymer as a Promising Carrier for Targeted Doxorubicin Delivery. PLoS ONE.

[17] Ralet M.C., Dronnet V., Buchholt H.C., Thibault J.F. (2001). Enzymatically and chemically de-esterified lime pectins: Characterisation, polyelectrolyte behaviour and calcium binding properties. Carbohydr. Res..

[18] Tolentino Chivite A. (2014). Ionic Complexes of Biodegradable Polyelectrolytes. Ph.D. Thesis.

[19] Abdelaal M.Y., Makki M.S.I., Sobahi Tariq R.A. (2012). Modification and Characterization of Polyacrylic Acid for Metal Ion Recovery. Am. J. Polym. Sci..

[20] Fang X., Somasundaran P. (2010). Swelling of Poly(acrylic acid) in Concentrated Sodium Carbonate Solution. J. Chem. Eng. Data.

[21] George M., Abraham T.E. (2006). Polyionic hydrocolloids for the intestinal delivery of protein drugs: Alginate and chitosan—A review. J. Control Release.

[22] Peppas N.A., Kavimandan N.J. (2006). Nanoscale analysis of protein and peptide absorption: Insulin absorption using complexation and pH-sensitive hydrogels as delivery vehicles. Eur. J. Pharm. Sci..

[23] Mundargi R.C., Rangaswamy V., Aminabhavi T.M. (2011). pH-Sensitive oral insulin delivery systems using Eudragit microspheres. Drug Dev. Ind. Pharm..

[24] Peppas N.A., Wood K.M., Blanchette J.O. (2004). Hydrogels for oral delivery of therapeutic proteins. Expert Opin. Biol. Ther..

[25] Alhaique F., Santucci E., Carafa M., Coviello T., Murtas E., Riccieri F.M. (1996). Gellan in sustained release formulations: Preparation of gel capsules and release studies. Biomaterials.

[26] Serra L., Doménech J., Peppas N.A. (2009). Engineering design and molecular dynamics of mucoadhesive drug delivery systems as targeting agents. Eur. J. Pharm. Biopharm..

[27] Kost J., Mathiowitz E. (1999). Intelligent drug delivery systems. Encyclopaedia of Controlled Drug Delivery.

[28] Bastakotin B.P., Liao S.-H., Inoue M., Yusa S.-I., Imura M., Nakashima K., Wu K.C., Yamauchi Y. (2013). pH-responsive polymeric micelles with Core-shell corona architectures as intracellular anti-cancer drug carriers. Sci. Technol. Adv. Mater..

[29] Xu C., Zhang J.S., Mo Y., Tan R.X. (2005). Calcium pectinate capsules for colon-specific drug delivery. Drug Dev. Ind. Pharm..

[30] Elliott J.E., Macdonald M., Nie J., Bowman C.N. (2004). Structure and swelling of poly(acrylic acid) hydrogels: Effect of pH, ionic strength, and dilution on the cross-linked polymer structure. Polymer.

[31] Itoh K., Kubo W., Fujiwara M., Watanabe H., Miyazaki S., Attwood D. (2006). The influence of gastric acidity and taste masking agent on in situ gelling pectin formulations for oral sustained delivery of acetaminophen. Biol. Pharm. Bull..

[32] Tian Y., Bromberg P.R., Hatton T.A., Tam K.C. (2007). Synthesis and aggregation behaviour of pluronic f87/polyacrylic acid block copolymer in the presence of doxorubicin. Langmuir.

[33] Zhang L., Eisenberg A. (1995). Multiple morphologies of “crew-cut” aggregates of polystyrene-b-poly(acrylic acid) block copolymers. Science.

[34] Iatridi Z., Tsitsilianis C. (2011). Water-Soluble Stimuli Responsive Star-Shape Segmented Macromolecules. Polymers.

[35] Pan D., Turner J.L., Wooley K.L. (2003). Folic acid-conjugated nanostructured materials designed for cancer cell targeting. Chem. Commun..

[36] Lee J.G., Kay E.P. (2006). FGF-2-induced wound healing in corneal endothelial cells requires Cdc42 activation and Rho inactivation through the phosphatidylinositol 3-kinase pathway. Investig. Ophthalmol. Vis. Sci..

[37] Laing B.M., Guo P., Bergstrom D.E. (2011). Optimized method for the synthesis and purification of adenosine—Folic acid conjugates for use as transcription initiators in the preparation of modified RNA. Methods.

[38] Banerjee S.S., Naval A., Patil R., Khandare J. (2012). Poly(ethylene glycol)-Prodrug Conjugates: Concept, Design, and Applications. J. Drug Deliv..

[39] Khoee S., Kavand A. (2014). A new procedure for preparation of polyethylene glycol-grafted magnetic iron oxide nanoparticles. J. Nanostruct. Chem..

[40] Sánchez-Márquez A., Fuentes-Ramírez R., Cano-Rodríguez I., Gamiño-Arroyo Z., Rubio-Rosas E., Kenny J.M., Rescignano N. (2015). Membrane Made of Cellulose Acetate with Polyacrylic Acid Reinforced with Carbon Nanotubes and Its Applicability for Chromium Removal. Int. J. Polym. Sci..

[41] Manrique G.D., Lajolo F.M. (2002). FT-IR spectroscopy as a tool for measuring degree of methyl esterification in pectins isolated from ripening papaya fruit. Postharvest Biol. Technol..

[42] Li J., Chen T., Deng F., Wan J., Tang Y., Yuan P., Zhang L. (2015). Synthesis, characterization, and in vitro evaluation of curcumin-loaded albumin nanoparticles surface-functionalized with glycyrrhetinic acid. Int. J. Nanomed..

[43] Kato T., Matsuoka T., Nishii M., Kamikawa Y., Kanie K., Nishimura T., Yashima E., Ujiie S. (2004). Supramolecular chirality of thermotropic liquid-crystalline folic acid derivatives. Angew. Chem. Int. Ed. Engl..

