# Supplementary material for: Polymeric Nano-Micelles as Novel Cargo-Carriers for LY2157299 Liver Cancer Cells Delivery
Source: Int J Mol Sci. 2018 Mar 6;19(3):748. doi: 10.3390/ijms19030748 (PMC5877609; doi:10.3390/ijms19030748)
Supplement: Supplementary file 1 [file ijms-19-00748-s001.pdf]

Supplementary Material

Polymers preparation, labeling and physical properties

The concentration of each used polymer are optimised after several experiments in our lab. Finally, 3 mg PgA was dissolved in 10 ml alkaline distilled water (pH; 10). 1 mg PAA was dissolved in 10 ml distilled water. 10 µl PEG-FA was dissolved in 10 ml distilled water. 200µl of Rhodamine (0.01mg/10 ml D.W) was added to 4.8 ml of PgA (3mg/10 ml). The properties of PgA and PAA were investigated at different pH.

Conjugation folic acid with polyethylene glycol

Folic acid conjugated with hydroxyl groups terminated PEG was performed by using procedure described in [16].

Folic acid activation

65 mg (0.15 mmol) Folic acid was dissolved in 2.5 ml dimethyl sulfoxide (DMSO) by overnight stirring, then 38 mg NHS (0.33 mmol) and 30 mg EDAC (0.17 mmol) were added into the solution to activate COOH groups of FA. The final molar ratio of FA/NHS/EDAC was 1: 2.2: 1.1.

Esterification

300 mg of hydroxyl group terminated PEG (0.15 mmol) was added to the solution in presence of EDAC. After 24 h, the product of reaction was dialyzed against distilled water for 1 week in a dialysis bag with a cut off MW of 1000, replacing water every 24 h.

Quantification of LY2157299 loaded nano-micelles by using Liquid chromatography coupled to mass Spectrometry (LC-MS)

LY loading into polymer nanomicelles (10µM/mL) was calculated by incubating LY solutions with known concentration overnight. The supernatant was removed by centrifugation and it was analysed by LC-MS. The loading percentage is defined as the residual LY moles in solution after loading divided by the moles of LY in solution before loading. In particular the encapsulation efficiency (% loading) is calculated as the relative difference between LY concentration before and after the incubation experiment (see. Equation: 1).

% Loading =100 X ([LY]i - [LY]f) /[LY]i (1)

[LY]i is defined as the initial concentration of LY. Liquid chromatography was performed using an HP 1200 system consisting of a binary pump, an automatic sampler, and a column oven (Agilent Technologies, Germany), which was equipped with an Poroshell 120 C18 column (Poroshell 120

SB-C18, 2.1 × 100 mm, 2.7 µm, Agilent, Milano, Italy). The column temperature was 25 °C. The mobile phase was composed of 0.1% formic acid in both (A) water and (B) acetonitrile (ACN). The isocratic elution was set at a flowrate of 0.3 mL/min. The run time was 10 min. An Agilent 6540 Accurate-Mass Quadruple Time-of-Flight with an electrospray ion source operated in positive mode was used for detection. Flow injection analysis was used to optimize the source parameters. The optimized source parameters for MS analysis were as follows: drying gas temperature 350 °C, gas flow 12 l/min, nebulizer gas flow pressure 35 psi, and capillary voltage 3,500 V. Data were acquired by Agilent Mass Hunter system (software version 6.0). The mass spectrometer was operated in full-scan mode in the m/z range 50–500. Extracted ion chromatograms (EICs) were obtained with an accu-racy of 10 ppm m/z from total ion chromatogram (TIC) employingthe m/z corresponding to [M+H]+ 370.1664. Peak areas were calculated employing the EICs. The calibration curve was constructed using five calibration standards (viz. 0.1-10 µM). The best fitting calibration curve was obtained by linear regression analysis with a 1/x weighting factor (R2 = 0.991).

Cell cultures and nano-micelles uptake

HLF were purchased by ATCC and were maintained in DMEM High Glucose (4,5g/l) supplemented with 5% L-glutamine, 10% fatal bovine serum, 5%penicillin-streptomycin and 5% sodium pyruvate in a humidified atmosphere of 37°C, 5% CO_2. HLF cells were seeded onto 4 sterilized coverslips, 20 000 cells for each one, sitting on the bottom of a multi-wells dish in the same culture conditions described above. After 24 hours, 100 µl of (2.3µM/1ml) LY2157299 loaded micelles, free micelles and free LY2157299 were added separately and incubated for 36 hours. Afterwards DMEM was removed. Cells were then fixed by 4% paraformaldehyde for 30 min. and successively washed in PBS at pH7.2. The cell permeabilization was done by using 0.1% Triton in PBS at pH7.2 for 3min. Non-specific binding of rabbit antibody was prevented by treatment with 5% BSA in PBS for 30min. SMA staining was performed according to manufacturer's protocol. Subsequently samples were incubated with an anti-mouse Alexa Fluor 488 conjugated secondary antibody (Cell Signaling Technology, USA). Coverslips were then removed carefully, and then cells spread onto coverslip were coated onto surface of clean slides by using a (4',6-diamidino-2-phenylindole) (DAPI)-mounting mixture for nuclear staining.

Supplementary Methods

Confocal laser scanning microscopy (CLSM)

The slides were analysed using a confocal laser scanning microscope (CLSM) (TCS SP5; Leica, Microsystem GmbH, Mannheim, Germany) equipped with a laser diode emitting at 405 nm, an argon-ion laser with a line at 488 nm, and a helium−neon laser for excitation at 543 nm.

The fluorescence signal of DAPI (blue channel) was detected after filtering with a band-pass filter within the range 415− 500 nm, the fluorescence signal of Alexa Fluor 488 SMA-secondary antibody (green channel) was detected with a band-pass filter 510−550 nm and the fluorescence signal of R6G conjugated micelles (red channel) was detected with a 560 nm long pass filter. Images were taken with a HCX PL APO lambda blue 63.0 x 1.40 oil-immersion objective.

Scanning electron microscopy

Sterilized 1mm of SiO_2 wafers were covered the bottom of multiwall surface then 500 cells were seeded up surface of SiO_2 wafer at 37 °C and 5% CO_2 then they incubated at 1 ml DMEM. Control of HLF and Cells were fixed in paraformaldehyde for 10 minutes, washed twice by PBS and dehydrated in series concentration of acetone (30%, 50%, 70%, 90% and 100%) then cells were dried and coated by a 5 nm gold layer. SEM measurements were conducted under 5 keV beam Energy by using SEM (JEOL JSM-6480LV). Images were processed by using dedicated software: SEM/JSM 500 instrument.

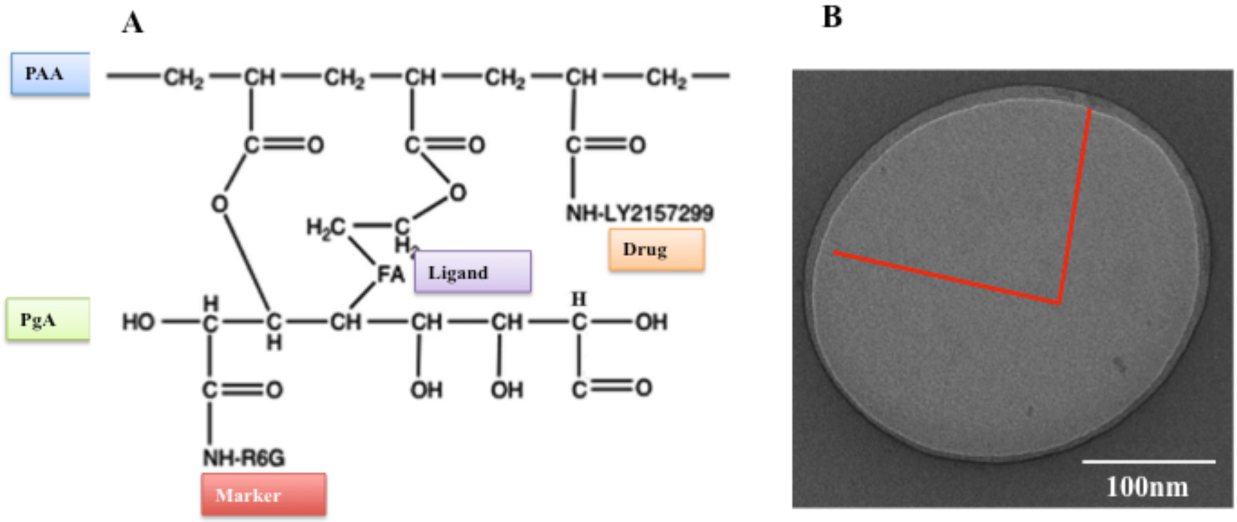

Scheme S1: Nano-micelles. A) Assembly structure of polyacrylic acid, polygalacturonic acid and folic acid-polyethylene glycol. B) TEM image of a nano-micelle.
